# Profiles of childhood adversities in Inuit from Nunavik: description and associations with indicators of socioeconomic characteristics, support, and community involvement

**DOI:** 10.17269/s41997-023-00750-z

**Published:** 2023-04-20

**Authors:** Anne-Julie Lafrenaye-Dugas, Richard E. Bélanger, Natalia Poliakova, Mylène Riva, Christopher Fletcher, Natacha Godbout, Sarah Fraser, Yohann Courtemanche, Caroline Moisan, Gina Muckle

**Affiliations:** 1 Population Health and Optimal Health Practices Axis, CHU de Québec – Université Laval Research Centre, Québec, QC Canada; 2 Département de pédiatrie, Faculté de Médecine, Université Laval, Québec, QC Canada; 3 McGill University, Montreal, QC Canada; 4 Université du Québec à Montréal, Montreal, QC Canada; 5 Université de Montréal, Montreal, QC Canada; 6 École de psychologie, Faculté des sciences sociales, Université Laval, Québec, QC Canada

**Keywords:** Adverse childhood experiences, Residential schools, Structural violence, Historical trauma, Inuit, Social determinants of health, Expériences négatives durant l’enfance, pensionnats autochtones, violence structurelle, traumatisme historique, Inuits, déterminants sociaux de la santé

## Abstract

**Objectives:**

Distress and associated health problems reported by Nunavik Inuit emanate from heterogeneous roots, including adverse childhood experiences. This study aims to (1) identify distinct childhood adversity profiles and (2) examine associations between these profiles and sex, socioeconomic characteristics, social support, and community involvement among Nunavimmiut.

**Methods:**

In a sample of 1109 adult Nunavimmiut, sex, socioeconomic characteristics, support, community involvement, residential school attendance, and 10 forms of adverse childhood experiences (ACEs) were documented using questionnaires. Latent class analyses and weighted comparisons were performed for three subgroups: 18–49 years; 50 years and above with experience of residential school; and 50 years and above without experience of residential school. The analysis design, the manuscript drafts, and the key findings were discussed and co-interpreted with the collaboration of community representatives, taking into consideration Inuit culture and needs.

**Results:**

A total of 77.6% of Nunavimmiut reported having experienced at least one form of childhood adversity. Three ACE profiles were identified among the 18–49-year-olds: low ACEs (43.0%), household stressors (30.7%), and multiple ACEs (26.3%). Two profiles characterized ACEs experienced among the 50-year-olds and over with and without history of residential schooling: low ACEs (80.1% and 77.2%, respectively) and multiple ACEs (19.9% and 22.8%, respectively). Among the group of 18–49-year-olds, as compared to the low ACE profile, the profile with household stressors included proportionally more women (odds ratio [OR] = 1.5) and was associated with lower involvement in volunteering and community activities (mean score reduced by 0.29 standard deviation [SD]) and lower family cohesion (SD = − 0.11), while the multiple ACE profile was related to a lower rate of employment (OR = 0.62), lower family cohesion (SD = − 0.28), and lower satisfaction with ability to practice traditional activities (SD = − 0.26).

**Conclusion:**

Childhood adversities among Nunavimmiut do not occur in isolation and experiencing multiple forms of childhood adversities predicts lower socioeconomic status, support, and community involvement in adulthood. Implications for the planning of health and community services in Nunavik are discussed.

**Supplementary information:**

The online version contains supplementary material available at 10.17269/s41997-023-00750-z.

## Résumé

**Objectifs:**

La détresse et les problèmes de santé associés rapportés par les Inuits du Nunavik émanent de racines hétérogènes, notamment des expériences négatives durant l’enfance. Cette étude vise à : 1) identifier des profils distincts d’adversités vécues durant l’enfance; et 2) examiner les associations entre ces profils et le sexe, les caractéristiques socioéconomiques, le soutien social et l’engagement communautaire des Nunavimmiut.

**Méthodes:**

Dans un échantillon de 1 109 adultes Nunavimmiut, le sexe, les caractéristiques socioéconomiques, le soutien, l’engagement communautaire, la fréquentation des pensionnats et 10 formes d’expériences négatives durant l’enfance (ENE) ont été documentés à l’aide de questionnaires. Des analyses de classes latentes et des comparaisons pondérées ont été réalisées pour trois sous-groupes : 18–49 ans; 50 ans et plus avec et sans expérience de pensionnat. Le plan d’analyses, les ébauches de manuscrit et les principaux résultats ont été discutés et co-interprétés avec la collaboration de représentants des communautés, en tenant compte de la culture et des besoins inuits.

**Résultats:**

Un total de 77,6 % des Nunavimmiut ont déclaré avoir vécu au moins une forme d’adversité durant l’enfance. Trois profils d’ENE ont été identifiés chez les 18–49 ans : ENE faibles (43,0 %), facteurs de stress domestiques (30,7 %) et ENE multiples (26,3 %). Deux profils caractérisaient les ENE vécus chez les 50 ans et plus avec et sans antécédents de pensionnat : faibles ENE (80,1 % et 77,2 %, respectivement) et ENE multiples (19,9 % et 22,8 %, respectivement). Parmi le groupe des 18–49 ans, par rapport au profil des ENE faibles, le profil avec facteurs de stress domestiques incluait proportionnellement plus de femmes (rapport de cotes [RC] = 1,5) et était associé à une plus faible implication dans le bénévolat et les activités communautaires (score moyen réduit de 0,29 écart-type [ET]) et une cohésion familiale plus faible (ET = -0,11), tandis que le profil d’ENE multiples était lié à un taux d’emploi plus faible (RC = 0,62), à une cohésion familiale plus faible (ET = -0,28) et à une plus faible satisfaction à l’égard de la capacité à pratiquer des activités traditionnelles (ET = -0,26).

**Conclusion:**

Les adversités vécues en enfance par les Nunavimmiut ne surviennent pas de manière isolée et le fait de vivre de multiples formes d’ENE prédit un statut socio-économique, un soutien et une implication communautaire inférieurs à l’âge adulte. Les implications pour la planification des services de santé et communautaires au Nunavik sont discutées.

## Introduction

Adverse childhood experiences (ACEs) describe any behaviour that may be harmful to the development or integrity of a child, such as sexual violence, psychological and physical abuse, physical or psychological neglect, and major household stressors such as witnessing violence, and having a family member attempting suicide, showing problematic substance use, mental illness, or being incarcerated (Centers for Disease Control & Prevention, 2022). ACEs rarely occur in isolation, and a child who underwent one form of violence presents higher risk of experiencing other forms. Also, in all populations, women have greater risk of experiencing sexual violence and of being subjected to several forms of violence than men (Haahr-Pedersen et al., 2020; Lafrenaye-Dugas et al., 2018; Lavoie et al., 2007).

The accumulation of ACEs is associated with health, psychosocial, and socioeconomic difficulties in adulthood (Haahr-Pedersen et al., 2020; Hodges et al., 2013). In turn, psychosocial and socioeconomic characteristics are strongly linked to mental and physical health (Brown et al., 2019; Haahr-Pedersen et al., 2020; Thoits, 2011). Several studies showed that certain specific patterns of childhood adversity experiences are associated with severe sequelae, and that distinct profiles of constellations of childhood adversities tend to form in populations at risk of violence and trauma (Brown et al., 2019; Haahr-Pedersen et al., 2020). For example, it has been documented that profiles comprising experiences of childhood sexual violence are particularly at risk of developing long-lasting health consequences (Debowska et al., 2017).

There are approximately 14,000 Inuit living in Nunavik, the northern region of the province of Quebec (Statistics Canada, 2017). They share with other Indigenous peoples a history of discriminatory and assimilative state policies, structural violence, and ongoing inequalities in access to resources and services (National Collaborating Centre for Indigenous Health [NCCIH], 2019, 2012). Thousands of Indigenous children were forced to leave their families for several years and to live in residential schools, set up by the Canadian government and run by the Church, where they were supposed to assimilate into the settlers’ culture and reject their cultural traditions (Bombay et al., 2014). Abuse and neglect were often experienced (Bombay et al., 2014; Truth and Reconciliation Commission of Canada [TRCC], 2015). In the Canadian North, the residential school system was implemented in an accelerated way, and thus created a sudden upheaval in the communities (TRCC, 2015). In 1949, 111 Inuit children were attending residential schools full-time in Canada, while in 1964 approximately 75% of school-aged Inuit attended them (David, 2006). In Nunavik, four residential schools were in operation from the mid-1950s until 1971 (George River [Kangirsualujjuaq; 1959–1960], Great Whale River [Kuujuaraapik/Whapmagoostui; 1960–1970], Payne Bay [Bellin, Kangirsuk; 1960–1962], and Port Harrison [Inukjuak; 1960–1971]; Bousquet, 2016; TRCC, 2015). Abuse and neglect were often experienced in such schools (Bombay et al., 2014; TRCC, 2015). Due to the separation from the family and the cultural disconnection, experienced residential schooling may also disrupt child-parent relations and family dynamics, with potentially significant impact on the residential school survivors’ experiences within their families and communities (Evans-Campbell, 2008). By consequence, the experience of childhood household adversities among older Inuit could vary according to residential school attendance. In addition, personal trauma, loss of culture, and traditional knowledge related to residential schooling tend to be intergenerationally transmitted (Bombay et al., 2014; First Nations Centre [FNC], 2005), suggesting even more frequent ACEs among descendants of former residential school students (Bombay et al., 2011).

ACEs are recognized as a major public health issue, as they are related to a vast range of long-lasting consequences in adulthood, such as mental health difficulties, physical health problems, sexual health issues, problematic substance use, re-experiencing violence in adulthood, and low socioeconomic status (SES) (Haahr-Pedersen et al., 2020; Norman et al., 2012; Widom et al., 2014). However, the experiences of different forms of childhood adversities and their effect on SES, support, and involvement in the community are insufficiently documented in the Inuit population of Nunavik, which limits the capacity of health professionals to offer comprehensive and appropriate services.

### Objectives

Since 2004, no general survey on Inuit health had been conducted in Nunavik. Thus, the actual study represents one step in making up for 15 years of absence of data on ACEs in that region. Our first objective is to compare the frequency of specific ACEs between Nunavik Inuit women and men, and between two generations, those who were children at the time of the residential schools and those born after the residential schools were closed. Second, we examine whether there are distinct profiles of ACEs accurately representing their prevalence, constellation, and diversity in Nunavik according to different sexes and age subgroups and, for Inuit aged 50 and over only, according to experienced residential schooling. Finally, within these subgroups, we test how adulthood socioeconomic characteristics, support, and community involvement are predicted by specific profiles of ACEs, experienced before age 18 and reported retrospectively.

## Methods

### Data collection

The Nunavik Health Survey 2017 (*Qanuilirpitaa? How are we now?*) originates from a resolution adopted by the Nunavik Regional Board of Health and Social Services (NRBHSS) demanding that a survey be organized to update the information on the health status of Nunavimmiut. *Qanuilirpitaa?* was conducted in partnership between all major Nunavik governmental, health and community organizations, the *Institut national de santé publique du Québec*, and academic researchers to provide up-to-date information on health and its determinants among Nunavik residents of two age groups, i.e., 16–30 years and 31 years and over. Consequently, the beneficiaries of the James Bay and Northern Québec Agreement residing in the 14 communities of Nunavik, aged 16 years and over, were eligible to participate. In pursuit of representativeness of the Nunavik population, a non-proportional, stratified, with replacement sampling strategy, based on sex, age group, and community, was used targeting a revised total sample of 1674 participants. Among individuals first identified as potential participants, only 36.5% were able to take part in the survey. Difficulties in contacting them or obstacles on their part due to weather, travelling, or scheduling issues are the main explanatory factors. A total of 1326 contributed to *Qanuilirpitaa?* (79.2% of the revised sample), from August 19 to October 5, 2017.

Our work is limited to participants aged 18 or older, since younger did not answer questions on ACEs. Thus, from a sample of 1266 participants having responded to the bloc of questions including those on ACEs, participants aged at least 18 were selected (*n* = 1148). People self-identifying as non-Inuit (*n* = 22), without information on residential school attendance (*n* = 7), and having responded to fewer than 6 ACE questions (*n* = 10) were excluded (final analytical sample *n* = 1109).

The *Qanuilirpitaa?* research protocol was approved by the ethics board of CHU de Québec-Université Laval. The recruiters were responsible to give a brief description of the survey and to show a short video on the consent form to participants, and to obtain their written consent to participate. Computerized questionnaires, pre-tested in a pilot study, were dispensed by trained Inuit and non-Inuit interviewers assigned individually to each participant depending on the participant’s language of choice (English, French, Inuktitut). Although interviewers were encouraged to dispense the entire questionnaire, if participants showed discomfort regarding a sensitive subject (e.g., violence) and sufficient reading skills, they could let the participants complete the questionnaires by themselves. The response rates for different blocks of questions ranged from 95.4% to 100%. The partial non-response rates for sensitive ACEs items specifically were between 0.7% and 2.3%, being slightly higher for women (maximum 2.5%) and younger participants (maximum 2.9%), while the non-response rate for the question on residential school attendance reached 2.1% (1.4% and 2.7% for men and women, respectively). For more information on the survey, see the methodological report of *Qanuilirpitaa?* by Hamel et al. (2020).

### Integrating Inuit and Indigenous perspectives and ethics

*Qanuilirpitaa?* and this study specifically were designed in full compliance with the ethics code developed to govern research within Indigenous people (ownership of, control of, access to, and possession of research processes, OCAP®; First Nations Information Governance Centre, 2014). An Inuit-led Steering Committee supervised the preparation, conduct, data interpretation, and diffusion of the survey results. A Data Management Committee (DMC) oversaw the construction of the battery of questionnaires, and approved data access requests. Thus, the proposal of this study, the data analysis plan, and the first manuscript draft were submitted to the *Qanuilirpitaa?* DMC, community representatives, and leaders, and were co-discussed in several in-person and online meetings throughout the realization of the project. The comments and suggestions shared by the committee and the regional partners were systematically applied and integrated into the manuscript. In a “two-eyed seeing approach” manner, the DMC and researchers co-interpreted the data taking into consideration Inuit culture and needs. The final manuscript and key findings were approved with the DMC.

### Measures

The scales and items used to assess the socioeconomic characteristics, support, and community involvement, the presence of ACEs, and residential school attendance are described in this section. Detailed information on these indicators is also available in Table S1 (Supplementary Material).

#### Adverse childhood experiences

The presence of ACEs (yes = 1/no = 0) before the age of 18 was retrospectively documented using the Adverse Childhood Experiences Questionnaire (ACE-Q; Felitti et al., 2019) in adult participants. The ACE-Q targets 10 specific experiences that can be grouped into four categories: (1) sexual violence, (2) abuse, (3) neglect, and (4) household stressors (see Table S1; Supplementary Material). Except for sexual violence, all the questions concerned events that had happened within the household. The accumulation of ACEs is represented by a total score out of 10 achieved by summing all affirmative answers. A cut-off of at least four forms of ACEs was also computed (Felitti et al., 2019). Since its first publication, the ACE-Q has been used in different populations, including Indigenous populations, and proven to be culturally sensitive (Zarse et al., 2019) and demonstrated a high internal consistency when used among other Indigenous populations (*α* = 0.78; Roh et al., 2015). Cronbach’s alpha shows adequate internal consistency in this study (0.77).

#### Residential schools

One item was used to assess direct experience of residential school (yes/no), defined in the questionnaire as “the residential school system attended by Aboriginal students”, among participants aged 50 years and over.

#### Socioeconomic characteristics, support, and community involvement

Socioeconomic characteristics include relationship status, education level, employment status, and income. Support and community involvement were represented by eight indicators grouped in three categories: perception of support, participation in social activities, and practice of traditional activities. A detailed description of these indicators is available in Table S1 (Supplementary Material) and in the associated report of *Qanuilirpitaa?* by Muckle and colleagues (2020).

### Data analysis

Descriptive analyses were performed on the weighted sample to compare the prevalence of ACEs between sexes and generations. Sample weights were used for all estimations and bootstrap replicate weights for variance estimation. The used weights considered the sample design and adjusted for the questionnaire non-response to ensure representativeness with the target population. The impact of partial non-responses for ACE questions (< 5%) was considered as negligible and was not adjusted for in descriptive and association analyses. Using Mplus 8.2 (Muthén & Muthén, 2017), latent class analysis (LCA) was used to identify distinct ACE profiles grounded on their similarities regarding each form of ACEs. A growing body of literature is available with examples of ACE data analyzed with LCA (e.g., Haahr-Pedersen, et al., 2020; Kim, et al., 2020). To select the optimal number of profiles, we examined the *Akaike’s Information Criterion* (AIC; Bozdogan, 1987) and the *Bayesian Information Criterion* (BIC; Schwarz, 1978), in addition to comparing the results to theoretical frameworks (Hair et al., 1998). Smaller AIC and BIC values indicate better model fit. Also, higher entropy represents better overall precision of group classification. Considering the different experiences of childhood adversities between the generations, clustering analyses were conducted separately among the 18−49-year-olds and the 50-year-olds and over. Since people who attended residential schools show specific characteristics (older age, lower levels of education, etc.), and the experience of residential schools is known as a distinctive traumatic experience, the 50 and over age group was divided into two subgroups: those who experienced residential schooling and those who did not. In the 18−49 age group, the 10 forms of ACEs were used to generate the clusters. In the 50-year-olds and over, the same forms of ACEs were used, except the item on separation or divorce of the parents considering its very low frequency in that age group (2.6%). Based on the LCA, profiles were described using posterior probability and variables indicating each participant’s profile membership were constructed. Chi-square tests and analyses of variance (ANOVAs) with post hoc comparisons were performed (a) to test differences on the distribution of forms of ACEs between classes and (b) to examine whether the profiles of ACEs are related to SES, support, and community involvement in adulthood, after controlling for sex. SAS survey procedures with bootstraps replicate weights were used for descriptive and comparison analyses (SAS Institute Inc., 2018). A bilateral alpha of 0.05 was used to compute all confidence intervals (Feise, 2002).

## Results

### Participants

Socioeconomic, support, and community involvement indicators are reported in Table 1. Half of the estimated population of Nunavik Inuit aged 18 years and over were men, 74.5% were aged 18–49 years, and 25.5% were aged 50 years or more. Within this group, approximately one third of people reported having attended residential school.

**Table 1:** Descriptive characteristics

Variables	Total sample *n*^1^ = 1109	18–49 years old *n*^1^ = 781	≥ 50 years old without residential schooling *n*^1^ = 216	≥ 50 years old with residential schooling *n*^1^ = 112	*p*-value comparing age groups^2^
	*M*/% (95% CI)	*M*/% (95% CI)	*M*/% (95% CI)	*M*/% (95% CI)	
Socioeconomic indicators					
Sex					
Men	49.1 (48.1, 50.2)	49.5 (47.9, 51.0)^a^	40.1 (34.7, 45.5)^b^	61.9 (53.9, 69.8)^c^	*F*(6.30), *p* = 0.002
Women	50.9 (49.8, 51.9)	50.5 (49.0, 52.1)	59.9 (54.5, 65.3)	38.1 (30.2, 46.1)	
Committed relationship					
Yes	56.5 (53.3, 59.7)	54.1 (50.1, 58.1)^a^	63.5 (57.3, 69.7)^b^	63.4 (53.0, 73.8)^a,b^	*F*(3.49), *p* = 0.03
No	43.5 (40.3, 46.8)	45.9 (41.9, 49.9)	36.5 (30.3, 42.7)	36.6 (26.2, 47.0)	
Education level					
Secondary school not completed	68.8 (65.7, 71.9)	65.6 (61.9, 69.4)^a^	81.6 (76.1, 87.2)^b^	73.4 (64.7, 82.1)^a,b^	*F*(8.82), *p* = 0.0002
Secondary school completed at least	31.2 (28.1, 34.3)	34.4 (30.6, 38.1)	18.4 (12.9, 23.9)	26.6 (17.9, 35.3)	
Employment					
Not working	28.6 (25.7, 31.5)	26.3 (22.9, 29.8)^a^	33.5 (26.2, 40.8)^a,b^	38.4 (28.9, 47.9)^b^	*F*(3.82), *p* = 0.02
Working	71.4 (68.5, 74.3)	73.7 (70.2, 77.2)	66.5 (59.3, 73.8)	61.6 (52.1, 71.1)	
Yearly income					
Under $20,000	56.9 (53.6, 60.1)	58.9 (54.9, 62.8)	49.6 (42.7, 56.6)	53.2 (42.9, 63.5)	*F*(2.62), *p* = 0.07
$20,000 or more	43.2 (39.9, 43.4)	41.1 (37.2, 45.1)	50.4 (43.4, 57.3)	46.8 (36.5, 57.1)	
Support and community involvement indicators					
Social support	13.4 (13.2, 3.6)	13.6 (13.3, 13.8)^a^	13.4 (12.8, 14.0)^a,b^	12.1 (11.3, 12.9)^c^	*F*(6.01), *p* = 0.003
Family cohesion	7.2 (7.1, 7.3)	7.1 (6.9, 7.2)^a^	7.5 (7.2, 7.9)^b^	7.6 (7.2, 8.0)^b,c^	*F*(4.74), *p* = 0.009
Community cohesion	11.7 (11.5, 11.9)	11.5 (11.3, 11.7)^a^	12.6 (12.3, 12.9)^b^	11.6 (11.1, 12.1)^a,c^	*F*(16.27), *p* < 0.001
Religious activities, at least monthly					
Less than monthly	58.2 (55.1, 61.3)	64.2 (60.5, 67.9)^a^	43.4 (35.8, 51.1)^b^	35.8 (25.8, 45.9)^b^	*F*(19.62), *p* < 0.0001
Monthly or more	41.8 (38.7, 44.9)	35.8 (32.1, 39.5)	56.6 (48.9, 64.2)	64.2 (54.1, 74.2)	
Volunteering and community activities	1.9 (1.8, 2.0)	1.8 (1.7, 1.9)^a^	2.1 (1.9, 2.3)^b^	2.1 (1.8, 2.3)^b^	*F*(4.47), *p* = 0.009
Healing and wellness activities					
No	71.7 (68.8, 74.7)	71.4 (68.1, 74.7)	72.5 (65.4, 79.6)	72.8 (64.4, 81.2)	*F*(0.08), *p* = 0.93
Yes	28.3 (25.3, 31.2)	28.6 (25.3, 31.9)	27.5 (20.4, 34.6)	27.2 (18.8, 35.6)	
Going on the land					
Less than often	56.1 (53.1, 59.0)	56.5 (52.7, 60.3)	54.8 (47.7, 61.9)	56.2 (45.6, 66.7)	*F*(0.09), *p* = 0.92
Monthly or more	43.9 (41.0, 46.9)	43.5 (39.7, 47.3)	45.2 (38.1, 52.3)	43.8 (33.3, 54.4)	
Satisfaction with ability to practice traditional activities	17.1 (16.9, 17.2)	16.9 (16.7, 17.0)^a^	17.8 (17.5, 18.1)^b^	17.4 (17.0, 17.8)^b^	*F*(15.52), *p* < 0.0001

Percentage and means on the same row with different superscript letters differ at *p* < 0.05

^1^Unweighted sample size. ^2^Wald log-linear chi-square test for categorical variables (*F*-test with 2/500 degrees of freedom) and ANOVA for continuous variables (*F*-test with 2 degrees of freedom)

### Description of ACEs

From the weighted sample, three out of four (77.6%) participants reported having experienced at least one form of ACEs, with an average of 2.6 forms (95% CI = 2.4–2.7) (data not shown in tables). Women reported a higher average number of ACEs (*M* = 2.8; 95% CI = 2.7–3.0) compared to men (*M* = 2.3; 95% CI = 2.1–2.6; *F* = 686.0; *p* < 0.001), as did younger Nunavimmiut (*M* = 2.9; 95% CI = 2.7–3.1) compared to older Nunavimmiut (*M* = 1.7; 95% CI = 1.4–1.9;* F* = 561.8; *p* < 0.001) (data not shown in tables). The prevalence of each form of ACEs was similar for women and men, except for sexual violence, living with someone presenting substance use, and living with someone presenting mental illness or suicidal behaviours, for which women reported higher prevalence. Inuit aged 50 and over reported significantly lower rates of all forms of ACEs compared to those aged 18 to 49, except for sexual violence and physical neglect, where no significant differences were observed. About a third of them indicated they attended the residential schools, with significantly more men than women reporting this experience (Table 2).

**Table 2:** Prevalence of forms of childhood adversity retroactively reported during adulthood

	Total *n*^1^ = 1109	Women *n*^1^ = 742	Men^2^ *n*^1^ = 367		18–49 years old *n*^1^ = 781	$\ge$ 50 years old^2^ *n*^1^ = 328		$\ge$ 50 years old with residential schooling *n*^1^ = 112	$\ge$ 50 years old without residential schooling^2^ *n*^1^ = 216	
	% (95% CI)	% (95% CI)	% (95% CI)	OR^3^ (95% CI)	% (95% CI)	% (95% CI)	OR^3^ (95% CI)	% (95% CI)	% (95% CI)	OR^3^ (95% CI)
Sexual violence	25.7 (22.9, 28.4)	**35.3 (31.8, 38.9)**	**15.7 (11.7, 19.7)**	**2.94 (2.06, 4.19)**	24.2 (20.9, 27.6)	29.8 (24.4, 35.1)	0.75 (0.54, 1.05)	28.9 (20.1 37.7)	30.3 (23.5, 37.0)	0.94 (0.53, 1.65)
Psychological abuse	32.7 (29.7, 35.8)	34.1 (30.6, 37.5)	31.3 (26.3, 36.4)	1.13 (0.84, 1.52)	**35.7 (32.9, 39.4)**	**24.0 (18.6, 29.5)**	**1.76 (1.23, 2.50)**	25.9 (17.2, 34.7)	22.9 (16.6, 29.2)	1.18 (0.65, 2.14)
Physical abuse	23.1 (20.2, 26.0)	24.1 (20.9, 27.2)	22.1 (17.4, 26.8)	1.12 (0.81, 1.55)	**26.2 (22.6, 29.8)**	**14.0 (10.1, 18.0)**	**2.18 (1.45, 3.26)**	13.8 (6.9, 20.6)	14.2 (9.2, 19.2)	0.96 (0.44, 2.11)
Psychological neglect	26.1 (23.1, 29.0)	28.8 (25.3, 32.2)	23.3 (18.5, 28.2)	1.33 (0.96, 1.84)	**28.3 (24.7, 31.9)**	**19.5 (15.0, 24.5)**	**1.60 (1.10, 2.33)**	19.6 (11.3, 28.0)	19.8 (14.0, 25.6)	0.99 (0.49, 1.98)
Physical neglect	17.4 (14.8, 20.0)	16.2 (13.5, 18.9)	18.6 (14.1, 23.1)	0.84 (0.58, 1.23)	18.2 (15.0, 21.4)	15.0 (10.7, 19.2)	1.27 (0.80, 2.02)	18.9 (10.7, 27.0)	12.6 (7.8, 17.5)	1.61 (0.72, 3.60)
Witnessing parents’ separation/divorce	23.6 (20.8, 26.4)	22.6 (19.7, 25.5)	24.7 (19.8, 29.6)	0.89 (0.64, 1.24)	**30.8 (27.0, 34.6)**	**2.6 (0.7, 4.4)**	**17.06 (4.60, 63.20)**	2.0 (0.0, 4.3)	2.9 (0.3, 5.4)	0.69 (0.04, 13.34)
Witnessing domestic violence against mother/stepmother	17.6 (15.1, 20.2)	20.5 (17.6, 23.3)	14.8 (10.8, 18.8)	1.48 (0.99, 2.21)	**20.2 (17.1, 23.3)**	**10.3 (6.7, 13.7)**	**2.19 (1.43, 3.36)**	9.4 (3.5, 15.3)	10.9 (6.8, 14.9)	0.85 (0.36, 2.04)
Living with someone presenting substance use	40.7 (37.6, 43.7)	**45.2 (41.5, 48.9)**	**35.9 (30.9, 48.9)**	**1.47 (1.12, 1.95)**	**47.7 (43.9, 51.6)**	**20.0 (15.2, 24.9)**	**3.64 (2.59, 5.13)**	15.5 (7.8, 23.2)	22.7 (16.6, 28.9)	0.62 (0.30, 1.30)
Living with someone presenting mental illness or suicidal behaviours	19.8 (17.0, 22.5)	**24.2 (20.9, 27.5)**	**15.2 (10.8, 19.6)**	**1.78 (1.17, 2.72)**	**22.6 (19.2, 26.0)**	**11.7 (7.5, 15.8)**	**2.21 (1.36, 3.60)**	10.7 (4.1, 17.3)	12.2 (6.9, 17.5)	0.86 (0.34, 2.20)
Living with someone who went to prison	34.3 (31.1, 37.5)	35.2 (31.6, 38.8)	33.4 (28.0, 38.7)	1.08 (0.80, 1.46)	**39.6 (35.6, 43.5)**	**19.0 (14.2, 23.7)**	**2.80 (1.96, 3.99)**	15.6 (8.1, 23.1)	20.9 (15.0, 26.9)	0.70 (0.35, 1.42)
At least one form	77.6 (74.8, 80.4)	80.0 (77.0, 83.0)	75.1 (70.5, 79.7)	1.33 (0.97, 1.81)	**82.3 (79.2, 85.4)**	**64.0 (57.8, 70.1)**	**2.61 (1.84, 3.72)**	65.5 (56.1, 74.9)	63.1 (55.5, 70.7)	1.11 (0.65, 1.89)
4 forms or more	30.8 (27.7, 33.9)	**34.0 (30.7, 37.3)**	**27.5 (22.4, 32.6)**	**1.36 (1.00, 1.84)**	**36.1 (32.1, 40.0)**	**15.4 (11.2, 19.6)**	**3.09 (2.10, 4.55)**	13.8 (7.3, 20.2)	16.4 (11.0, 21.8)	0.81 (0.40, 1.65)
Residential school attendance (among those 50 years and over)	-	**27.3 (20.4, 34.2)**	**47.6 (38.5, 56.8)**	**0.41 (0.24, 0.71)**	-	37.1 (31.2, 43.0)	-	-	-	-

*OR* odds ratios, *CI* confidence interval

^1^Unweighted sample size. ^2^Reference group. Bolded values indicate significant differences. ^3^Wald log-linear chi-square test

### ACE profiles description

Models up to five classes were examined for each of the three groups. Fit statistics and classification coefficients of all models are reported in Table S2 (Supplementary Material).

**Fig. 1:**
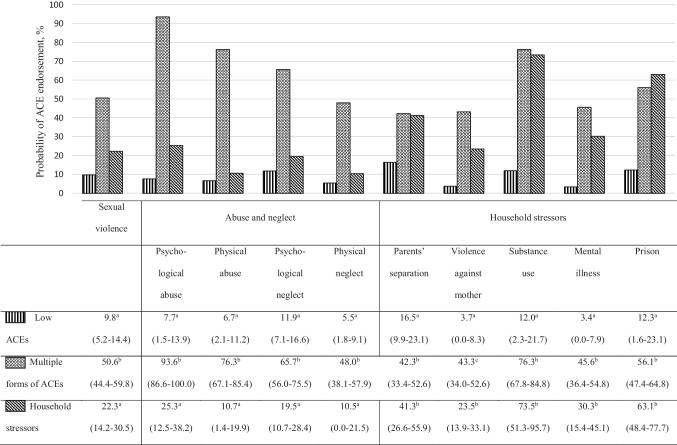
Plot of latent classes with their relative prevalence and posterior probabilities (with 95% confidence interval) of adverse childhood experiences for the 18−49-year-olds. Note: Percentages on the same column with different superscript letters differ at *p* < 0.05

In the 18−49-year-old group, three profiles were identified (see Fig. 1). The most prevalent profile entitled “low ACEs” (43.0%) is characterized by the lowest probability of any form of ACEs. On average, Nunavimmiut in “low ACEs” profile presented 0.8 (95% CI = 0.7–0.9) forms of ACEs, and 39.7% did not report any ACE. Participants in the “multiple forms of ACEs” profile (30.7%) reported the highest rate of all forms of ACEs, an average of 6.0 forms of ACEs, and they all showed at least two forms of ACEs. The “household stressors” profile (26.3%) was mainly characterized by exposure to many household stressors, especially a household member experiencing substance use and going to prison. On average, individuals in this profile reported 3.3 forms of ACEs, and they all experienced at least one form of ACEs. The “low ACEs” profile comprised lower rates of women (OR = 0.65; 95% CI = 0.44–0.96; *p* = 0.030) and lower rates of younger participants aged 18–29 (OR = 0.64; 95% CI = 0.43–0.94; *p* = 0.021) than the “household stressors” profile. Participants from the “low ACEs” profile were significantly more likely to be in a committed relationship (OR = 1.74; 95% CI = 1.18–2.56; *p* = 0.005) and to be working and employed (OR = 1.60; 95% CI = 1.01–2.53; *p* = 0.035) compared to the “multiple forms of ACEs” profile. Participants in the “low ACEs” profile reported a higher level of family cohesion of 0.28 standard deviation (SD; 95% CI = 0.18–0.38) than those in the “multiple forms of ACEs” and than those in the “household stressors” profile (SD = 0.11; 95% CI = 0.01–0.21). Participants in the “multiple forms of ACEs” reported lower levels of community cohesion than those in the “low ACEs” (SD = − 0.31; 95% CI = − 0.48 to − 0.14) and those in the “household stressors” (SD = − 0.22; 95% CI = − 0.41 to − 0.03) profiles. Participants in the “low ACEs” profile were more involved in volunteering and community activities (SD = 0.29; 95% CI = 0.04‒0.54) than the “household stressors” profile and more satisfied with their ability to practice traditional activities (SD = 0.26; 95% CI = 0.10‒0.43) than those in the “multiple forms of ACEs” profile (Table 3).

**Table 3:** Association of profiles with indicators of socioeconomic status, support, and community involvement in the 18–49 subgroup (chi^2^, ANOVA, and post hoc analyses)

Socioeconomic indicators	Low ACEs % (95% CI)	Multiple forms of ACEs % (95% CI)	Household stressors % (95% CI)	*P*^1^
Sex				
Women	45.0 (40.4–49.6)^a^	54.3 (47.6–61.0)^a,b^	55.7 (49.0–62.3)^b^	*F*(3.14), *p* = 0.04
Men	55.0 (50.4–59.6)	45.7 (39.0–52.4)	44.3 (37.7–51.0)	
Age subgroups				
18–29-year-olds	45.5 (40.7–50.2)^a^	48.6 (41.6–55.6)	56.6 (49.9–63.2)^b^	*F*(5.22), *p* = 0.06
30–49-year-olds	54.5 (49.8–59.3)	51.4 (44.4–58.4)	43.4 (36.8–50.0)	
Committed relationship				
Yes	59.4 (53.6–65.1)^a^	45.7 (38.1–53.3)^b^	53.6 (46.0–61.3)^a,b^	*F*(7.81), *p* = 0.02
No	40.6 (34.9–46.4)	54.3 (46.7–61.9)	46.4 (38.7–54.0)	
Education level				
Secondary school not completed	67.1 (61.4–72.7)	68.3 (61.2–75.5)	61.0 (53.6–68.4)	*F*(2.31), *p* = 0.32
Secondary school completed at least	32.9 (27.3–38.6)	31.7 (24.5–38.8)	39.0 (31.6–46.4)	
Employment				
Not working	23.6 (18.4–28.8)^a^	33.0 (25.9–40.2)^b^	24.5 (18.7–30.3)^a^	*F*(5.46), *p* = 0.07
Working	76.4 (71.2–81.6)	67.0 (59.8–74.1)	75.5 (69.7–81.3)	
Yearly income				
Under $20,000	56.1 (50.0–62.2)	58.4 (51.0–65.7)	63.7 (56.2–71.1)	*F*(2.44), *p* = 0.30
$20,000 or more	43.9 (37.8–50.0)	41.6 (34.3–49.0)	36.3 (28.9–43.8)	
Support and community involvement indicators*	*M*/% (95% CI)	*M*/% (95% CI)	*M*/% (95% CI)	
Perception of support				
Social support	13.6 (13.2–14.1)	13.3 (12.8–13.8)	13.7 (13.1–14.2)	*F*(0.54), *p* = 0.59
Family cohesion	7.5 (7.3–7.7)^a^	6,4 (6.0–6.7)^b^	7,1 (6.7–7.4)^a,c^	*F*(15.5), *p* < 0.001
Community cohesion	11.8 (11.5–12.1)^a^	10.9 (10.5–11.3)^b^	11.6 (11.2–11.9)^a,c^	*F*(6.32), *p* = 0.002
Participation in social activities				
Religious activities, at least monthly	36.6 (31.0–42.2)	35.6 (28.8–42.4)	34.7 (27.7–41.7)	*F*(0.10), *p* = 0.90
Volunteering and community activities	4.1 (3.8–4.3)^a^	3.8 (3.5–4.1)	3.7 (3.4–3.9)^b^	*F*(2.76), *p* = 0.07
Healing and wellness activities, yes	28.2 (23.2–33.2)	31.0 (24.3–37.7)	26.9 (20.9–32.9)	*F*(0.83), *p* = 0.44
Practice of traditional activities				
Going on the land, often	43.8 (37.7–49.9)	40.9 (33.9–48.0)	45.4 (37.9–52.8)	*F*(0.38), *p* = 0.68
Satisfaction with ability to practice traditional activities	17.1 (16.8–17.3)^a^	16.5 (16.2–16.8)^b^	16.9 (16.6–17.2)^a,b^	*F*(4.98), *p* = 0.007

*Note:* Percentage and means on the same row with different superscript letters differ at *p* < 0.05

*M* mean, *CI* confidence interval

^*^The support and community involvement indicators are controlled for sex

^1^Wald log-linear chi-square test for categorical variables (*F*-test with 2/500 degrees of freedom) and ANOVA for continuous variables (*F*-test with 2/500 degrees of freedom)

**Fig. 2:**
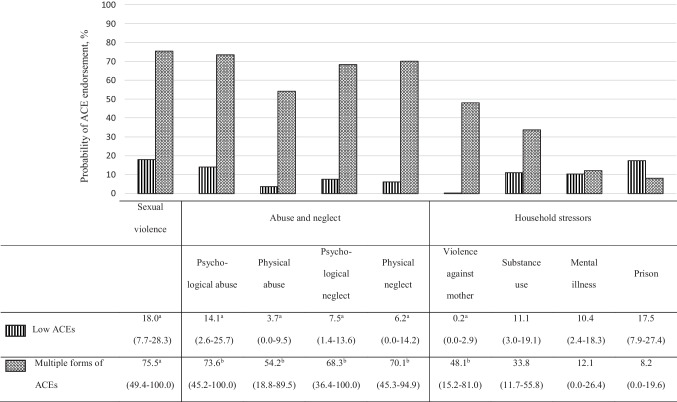
Plot of latent classes with their relative prevalence and posterior probabilities (with 95% confidence interval) of adverse childhood experiences for the 50-year-olds and over who experienced residential schooling. Note: Percentages on the same column with different superscript letters differ at *p* < 0.05

For those 50 years and over who experienced residential schooling, two profiles emerged (see Fig. 2). Participants in the “low ACEs” (80.1%) profile demonstrated the lowest prevalence of all forms of ACEs, except for having lived with a household member who exhibited substance use or mental health issues or who went to prison, for which the “multiple forms of ACEs” profile (19.9%) revealed similar estimated probability of endorsement. Respondents in the “low ACEs” profile reported an average number of 0.92 (95% CI = 0.70–1.13) forms of ACEs, and 42.3% (95% CI = 31.3–53.4) of them did not report any ACE. The “multiple forms of ACEs” reported high rates of ACEs. On average, people included in this profile reported 4.6 (95% CI = 4.0–5.2) forms of ACEs, and they all reported having undergone at least one form of ACEs. The two profiles did not stand out on any of the socioeconomic or support and community involvement indicators (Table 4).

**Table 4:** Association of profiles with indicators of socioeconomic status, support, and community involvement in the 50 and over subgroup (chi^2^, ANOVA, and post hoc analyses)

	With residential schooling				Without residential schooling		
Socioeconomic indicators	Low ACEs % (95% CI)	Multiple forms of ACEs % (95% CI)	*P*^2^		Low ACEs % (95% CI)	Multiple forms of ACEs % (95% CI)	*P*^2^
Sex (%)							
Women	34.7 (25.4–43.90)	53.4 (31.6–75.1)	*F*(2.23), *p* = 0.14		58.1 (51.4–64.7)	66.0 (52.3–79.7)	*F*(0.89), *p* = 0.35
Men	65.3 (56.1–74.6)	46.6 (24.9–68.4)			41.9 (35.3–48.5)	34.0 (20.3–47.7)	
Committed relationship (%)							
Yes	66.2 (54.7–77.7)	50.9 (29.6–72.1)	*F*(1.73), *p* = 0.19		64.8 (57.8–71.7)	59.1 (45.1–73.0)	*F*(0.56), *p* = 0.46
No	33.8 (22.2–45.3)	49.1 (27.9–70.4)			35.2 (28.3–42.2)	40.9 (27.0–54.9)	
Education level (%)							
Secondary school not completed	73.1 (63.2–82.9)	74.7 (55.2–94.2)	*F*(0.02), *p* = 0.89		83.3 (77.2–89.5)	75.8 (62.3–89.2)	*F*(1.06), *p* = 0.30
Secondary school completed at least	26.9 (17.1–36.8)	25.3 (5.8–44.8)^1^			16.7 (10.5–22.8)	24.2 (10.8–37.7)^1^	
Employment (%)							
Not working	38.6 (27.8–49.4)	37.6 (15.5–59.6)^1^	*F*(0.01), *p* = 0.93		34.7 (26.3–43.1)	29.1 (15.7–42.6)	*F*(0.47), *p* = 0.49
Working	61.4 (50.6–72.2)	62.4 (40.4–84.5)			65.3 (56.9–73.7)	70.9 (57.4–84.3)	
Yearly income (%)							
Under $20,000	52.9 (41.3–64.5)	54.5 (31.9–77.2)	*F*(0.02), *p* = 0.89		46.4 (38.0–54.9)	60.8 (46.3–75.3)	*F*(2.51), *p* = 0.11
$20,000 or more	47.1 (35.5–58.7)	45.5 (22.8–68.1)^1^			53.6 (45.1–62.0)	39.2 (24.7–53.7)	
Support and community involvement indicators*	*M*/% (95% CI)	*M*/% (95% CI)	*p*^2^		*M*/% (95% CI)	*M*/% (95% CI)	*p*^2^
Perception of support							
Social support	12.3 (11.5–13.1)	12.2 (10.1–14.3)	*F*(0.00), *p* = 0.95		13.4 (12.7–14.1)	12.6 (11.4–13.8)	*F*(1.58), *p* = 0.21
Family cohesion	7.7 (7.2–8.2)	6.9 (5.9–7.9)	*F*(2.14), *p* = 0.43		7.7 (7.4–8.0)	7.2 (6.2–8.1)	*F*(1.13), *p* = 0.29
Community cohesion	11.7 (11.1–12.3)	11.0 (9.7–12.4)	*F*(0.63), *p* = 0.43		12.7 (12.4–13.0)	12.3 (11.5–13.0)	*F*(0.98), *p* = 0.33
Participation in social activities							
Religious activities, at least monthly	63.1 (51.9–74.7)	68.8 (45.6–92.0)	*F*(0.17), *p* = 0.68		57.1 (48.5–65.7)	54.7 (39.3–70.2)	*F*(0.07), *p* = 0.79
Volunteering and community activities	4.5 (4.1–5.0)	4.0 (2.7–5.3)	*F*(0.61), *p* = 0.44		4.3 (3.9–4.7)	5.0 (4.4–5.6)	*F*(3.56), *p* = 0.06
Healing and wellness activities, yes	27.2 (17.7–36.7)	26.9 (9.4–44.5)^1^	*F*(0.00), *p* = 0.98		24.9 (17.2–32.6)	36.6 (22.1–51.1)	*F*(2.33), *p* = 0.13
Practice of traditional activities							
Going on the land, often	47.1 (35.6–58.7)	29.2 (9.0–49.5)^1^	*F*(2.14), *p* = 0.14		44.0 (35.8–52.2)	49.6 (35.0–64.1)	*F*(0.41), *p* = 0.52
Satisfaction with ability to practice traditional activities	17.5 (17.0–17.9)	17.3 (16.1–18.5)	*F*(0.08), *p* = 0.79		17.8 (17.5–18.2)	17.8 (17.2–18.4)	*F*(0.00), *p* = 0.99

Percentage and means on the same row with different superscript letters differ at *p* < 0.05

*M* mean, *CI* confidence interval

^*^The support and community involvement indicators are controlled for sex

^1^The coefficient of variation is greater than 25%. The proportion should be interpreted carefully

^2^Wald log-linear chi-square test for categorical variables (*F*-test with 1/500 degrees of freedom) and ANOVA for continuous variables (*F*-test with 1/500 degrees of freedom)

**Fig. 3:**
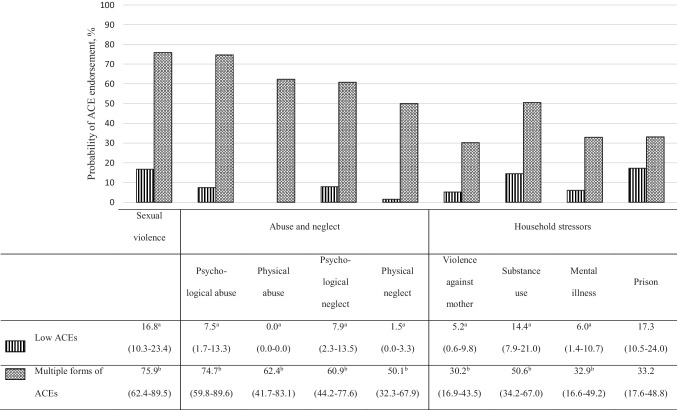
Plot of latent classes with their relative prevalence and posterior probabilities (with 95% confidence interval) of adverse childhood experiences for the 50-year-olds and over who did not experience residential schooling. Note: Percentages on the same column with different superscript letters differ at *p* < 0.05

In the group aged 50 years and more who did not experience residential schooling, two profiles emerged (see Fig. 3). The “low ACEs” profile (77.2%) is characterized by the lower prevalence of all ACEs, except for having lived with a household member who went to prison, presenting a similar estimated probability of endorsement for this ACE with the “multiple forms of ACEs” profile (22.8%). People in this “low ACEs” profile reported having experienced an average of 0.8 (95% CI = 0.6–0.9) forms of ACEs, and 47.4% (95% CI = 38.5–56.4) did not report any ACE. Participants from the “multiple forms of ACEs” profile showed an average number of 4.8 (95% CI = 4.4–5.3) forms of ACEs and they all reported at least one form of ACEs. The two profiles did not differ on any of the socioeconomic or support and community involvement indicators.

## Discussion

In the current study, we observe high rates of ACEs among the Inuit population of Nunavik, with prevalence varying by age and sex. It was postulated that exploring specific profiles of constellations of childhood adversities could lead to a deeper understanding of these experiences and their repercussions, comparatively to studying an isolated form of adversity, or a single cumulative score of adversities. Thereby, the findings also highlighted the heterogeneity in the experiences of ACEs in that population, with ACEs clustering to form specific profiles, which are in turn distinctively associated with indicators of SES, support, and community involvement later in adulthood. These data were analyzed and interpreted in collaboration with partner organizations and the DMC. They reviewed the different versions of the manuscript, and work sessions were organized. The co-interpretation process focused mainly on the higher rates of ACEs found among the 18–49 generation compared to the older generation, the high rates of sexual violence among participants aged 50 and older who did not attend residential schools, the long-term impacts of residential schools and ACEs, and some limitations of the questionnaires and methods.

Three out of four participants (77.6%) indicated having experienced at least one form of ACEs. In comparison, a study conducted among Greenlandic Inuit and assessing three forms of ACEs (alcohol problems in childhood home, physical violence, and sexual violence) described that 66.0% reported at least one form of ACEs (Bjerregaard & Larsen, 2018). Targeting the Canadian population in general, the Canadian Longitudinal Study of Aging (the 2015–2018 follow-up) revealed that 61.6% of participants aged 45–85 years had experienced before age 16 at least one of eight ACEs targeted by the short form of the Childhood Experiences of Violence Questionnaire (Joshi et al., 2021). Elsewhere, using data from the 2012 Canadian Community Health Survey – Mental Health, prevalence of at least one of six early-childhood adversities experienced before age 16 was estimated at 49.6% among Canadians aged 20 years and older (Baiden et al., 2015). Even if no comparison can be drawn for different reasons (e.g., different age groups and questionnaires used), our analyses suggest relatively high rates of childhood adversities within Inuit of Nunavik.

More specifically, our results showed that women reported a higher number of different forms of ACEs compared to men, which is consistent with current literature. We also observe an over-representation of women in profiles exhibiting the highest level of victimization. In Indigenous populations as in non-Indigenous populations, studies pointed out that girls and women tend to be more at risk of experiencing adverse events and their long-term consequences on health than boys and men (Haahr-Pedersen et al., 2020; Lafrenaye-Dugas et al., 2018; Lavoie et al., 2007).

Participants aged 18 to 49 reported more forms of ACEs than those aged 50 and over. One possible explanation could be that significant changes in the structures and dynamics of Inuit families such as a decrease in closeness and an increase in maltreatment were triggered by the introduction of assimilative and discriminatory measures by settler governments (Kral, 2016). Discriminatory and violent measures such as residential schooling had impacts not only on individuals, but also on communities, by reducing their sense of empowerment and ability to address their well-being and healing, and by intensifying the phenomenon of intergenerational transmission of trauma (Suicide Prevention Strategy Working Group, 2010; TRCC, 2015). Having attended residential schools is known as a risk factor for substance use, and it is documented that problematic substance use has been increasing in Nunavik since these schools closed (Fortin et al., 2015; Ross et al., 2015). In turn, Indigenous people whose parents or grandparents attended residential schools indeed show a greater risk of experiencing maltreatment (Bombay et al., 2014; Elias et al., 2012). Furthermore, despite Inuit aged 50 and over showing lower exposure to ACEs, they were at risk of undergoing structural traumas and adversities outside the household, considering that one out of three reported residential school attendance.

Among the 18–49-year-olds, the data reveal that women from the “multiple forms of ACEs” profile were less likely to have access to social support. Violence against girls and women raises concerns for many Indigenous communities in Canada, considering that Indigenous women from Canada are more likely to suffer from abuse than non-Indigenous Canadian women (Andersson & Nahwegahbow, 2010; Elias et al., 2012). These results highlight the importance of offering services targeting Inuit women and their specific health needs, and providing them with enough safe spaces.

Still in the 18–49-year-olds, participants pictured in the “multiple forms of ACEs” profile reported the lowest commitment in a relationship and employment rates, which is consistent with discussions with Inuit collaborators, as well as data documented in the existing literature (Gone, 2013), highlighting the association between adversities and difficulty in adulthood with maintaining healthy romantic relationships and stable employment. The “multiple forms of ACEs” profile also reported the lowest scores of family cohesion, community cohesion, and satisfaction with ability to practice traditional activities, which suggests that a concomitant accumulation of abuse, neglect, and household stressors is associated with difficulties in connecting with one’s family, community, and culture. Likewise, the “household stressors” profile showed less participation in volunteering and community activities, which highlights that an accumulation of stressful events in the home is linked to difficulties in getting involved in social activities. In general, these results suggest that the accumulation of several forms of ACEs is associated many years later with a lower likelihood to be an active member of the community. In line with the literature (Gone, 2013; Haahr-Pedersen et al., 2020), exchanges on the results with Inuit representatives support the idea that an individual having faced ACEs could benefit from services intervening directly on family and social cohesion and on connection with cultural and spiritual traditions, allowing them to break the isolation in which many survivors of violence live.

Among the groups of 50-year-olds and over who experienced residential schooling and who did not, we also find a “low ACEs” profile and a “multiple forms of ACEs” profile. However, the “multiple forms of ACEs” profile among Nunavimmiut having experienced residential schooling is characterized mainly by exposure to sexual violence and childhood abuse and neglect, while among those without residential school experiences, the same profile differs from the “low ACEs” profile on all ACEs, except for having lived with a household member who went to prison. For both groups, the profiles do not differ in terms of socioeconomic characteristics, support, and community involvement. Different points are worth highlighting in relation to these results. First, taking into consideration that many Nunavimmiut aged 50 years and older are now deceased, survivors could not be perfectly representative of this generation, which could explain the small variability on the socioeconomic, support, and community involvement indicators in these groups. Selection bias by death is particularly relevant with regard to the group having experienced residential schooling because this group is the oldest of our sample but also because of known deleterious effects of residential school attendance on health and well-being (Wilk et al., 2017). Second, weaker associations between ACEs and later health and social outcomes with increasing age have also been documented previously (e.g., Atkinson et al., 2023). With advancing age, adulthood life events and health factors unrelated to childhood exposure can intervene and moderate ACE effects. In addition, the groups of 50-year-olds are relatively small. A small sample size could limit statistical power and prevent from identification of smaller classes and of significant associations with outcome indicators (e.g., Nylund-Gibson & Choi, 2018). Wider confidence intervals of results obtained especially for the group having experienced residential schooling would support this potential explanation for the results. Also, the used ACE questionnaire does not make a distinction between sexual violence experienced in the household and that experienced outside of the family. By consequence, both within the group with history of residential schooling and without, experience of ACEs could be more heterogeneous, with potentially undiscovered smaller ACE profiles. Finally, it should also be noted that the three age groups grew up in sociocommunity contexts that may not be directly comparable, which may have coloured the participant’s answers and could explain the differences in the profiles’ distribution of ACEs from one age group to another.

Latent class analyses revealed presence of distinct ACE profiles within the group aged 18–49. Also, the “multiple forms of ACEs” profiles among the groups aged 50 years and over with and without history of residential school attendance differed with regard to prevalence of household stressors. These results are congruent with previous ACE literature suggesting frequent co-occurrence of ACEs within different general and clinical populations where ACE co-occurrence frequently results in clustered patterns in two domains, namely childhood maltreatment and household stressors that present differential association with socioeconomic and sociocultural indicators (e.g., Barboza, 2018; Elm et al., 2021; Grest et al., 2021). Despite marked variability in profile associations with adulthood outcomes, our results and previous studies highlight that the “multiple forms of ACEs” profile presents higher risk of multiple negative effects in adulthood. Also, among all three groups, this profile is characterized by high probability of endorsement of sexual violence. These results suggest that sexual violence was present in the communities of Nunavik outside of the residential schools, and that the household environment found in these profiles seems to be also associated with higher risk of sexual violence. It is widely documented that sexual violence is associated with several health issues (e.g., problematic sexual behaviours, gynecological and obstetric difficulties, psychiatric difficulties, self-harming behaviours), especially when occurring in the context of a profile cumulating multiple other forms of adversity (American College of Obstetricians and Gynecologists, 2011; Debowska et al., 2017). Additionally, since women are more at risk of experiencing sexual violence than men (Haahr-Pedersen et al., 2020; Lavoie et al., 2007), this places women more at risk for negative adulthood health outcomes (Westfall & Nemeroff, 2015). Knowledge about groups with similar experience and at higher risk for adult adverse consequences can be informative for communities to identify the most vulnerable subgroups of the population. Also, even if there is a significant knowledge gap on effective interventions to mitigate ACEs negative impact, some data suggest that the groups with common ACE experiences may benefit from similar interventions (Lorenc et al., 2020). Previous studies demonstrated variability in outcomes among adults with high levels of ACEs (Kalmakis & Chandler, 2015), highlighting a non-deterministic nature of ACEs experience and individual- and community-level capacity to mobilize adaptive processes (Masten, 2021). With regard to Indigenous populations, studies recommend, for example, that residential school survivors who were deprived of their traditions during their childhood due to separation from their families have access to resources focusing on culturally grounded interventions (Gone, 2013; Rowan et al., 2014). Allowing them to share with peers who endured similar experiences is also recognized as a helpful healing approach (Gone, 2013; Rowan et al., 2014).

### Limitations of the study and avenues for future research

The conclusions of this study should be moderated in consideration of its limitations. First, self-reported questionnaires are subject to a recall bias, and the ACE-Q does not allow an assessment of the ACEs severity nor their traumatic repercussions. The subgroup who experienced residential schooling being significantly older than the other subgroups, this bias could have particularly affected their ability to answer the questionnaires. Second, since interviewers were present for the completion of the questionnaires, social desirability bias may have influenced the responses of participants, for example, by reducing the comfort of disclosing ACEs. Third, despite the high response rate to the questions assessing ACEs, approximately one third of the Nunavimmiut first identified as potential participants ended up completing the survey. Nunavimmiut who answered the survey might therefore differ from non-respondents in terms of experienced ACEs. Fourth, except for sexual violence, all the questions about ACEs are related to events that occurred within the household, and no question directly documented violence experienced in residential schools. Fifth, in the Canadian North, residential schools were not the only assimilative schools, and a Federal Day Schools system was also in place. However, the questionnaire only asked about residential schools, while many Inuit from Nunavik attended Federal Day Schools, so we cannot rule out that some respondents understood the question as referring to both residential schools and Federal Day Schools. In addition, the questions about physical and psychological abuse and neglect targeted ACEs that occurred in the home environment, excluding adversities people may experience outside the household. This could result in an underestimation of ACEs’ prevalence and diversity, and their associations with socioeconomic, support, and community involvement indicators. Taking into consideration a limited scope and other limits of the ACE questionnaire (McLennan et al., 2020), future studies would benefit from studying other adverse experiences, both specific and non-specific to the Inuit history, such as the sled dogs slaughter, the relocation of Inuit families on High Arctic islands, or childhood exposure to poverty. Sixth, previous studies have highlighted an intergenerational effect of parental residential school attendance on ACEs among subsequent generations (e.g., Bombay et al., 2011). This effect was not taken into consideration in this study and could affect the results. Seventh, even if the last residential school in Nunavik closed in 1971, it is possible that participants under 50 years of age not questioned about experience of residential schooling may have attended residential school. As a result, this could underestimate the prevalence of residential school attendance and lead to more similar results in the groups with and without experience of residential schooling. Eighth, associations between identified profiles and socioeconomic, support, and community involvement indicators could be affected by age of participants (especially for the 18–49 group characterized by a broad range of ages among participants) as some effects of ACE exposure may take a longer time period to manifest. As our results on ACE profiles lead to a better understanding of the experiences of childhood adversities among Nunavimmiut, it would be relevant for future studies to determine whether and how these distinct profiles are related to mental, sexual, and physical health outcomes in adulthood. Other studies should also explore the experience of abuse and neglect within the residential schools, as well as the long-term repercussions of structural violence and ACEs on the well-being of Inuit communities in addition to analyses at the individual level.

## Conclusion

By linking ACEs, their clustering as profiles, residential school attendance, and contemporary predictors of health, this study provides a better understanding and evidence of how these early life experiences are shaping long-term life trajectories and thereby, overall health. The Inuit population of Nunavik reports high rates of ACEs, combined with a collective history of systemic violence. Some people appear particularly at risk of cumulating many forms of violence and suffering long-term social consequences. Health and community services rooted in their needs and realities could help reduce the transmission of trauma from one generation to the next, while allowing them to reclaim their cultural heritage. In addition to guiding the public health decision-making process regarding childhood adversities, their long-term impacts, and intergenerational transmission of trauma (Haahr-Pedersen et al., 2020), our results highlight the importance of access to trauma-informed care and culturally safe care in Nunavik (Browne et al., 2016). These approaches aim to recognize the multiple short-term and long-term effects of violence on health and social integration, the consequences of past and present interpersonal and systemic violence, and the importance of anchoring health care in the culture of service users (Browne et al., 2016).

## Contributions to knowledge

What does this study add to existing knowledge?

- This study fills 15 years of lack of data on childhood adversities in Nunavik.
- The data show that Nunavimmiut report experiencing several different forms of childhood adversity that tend to cluster and form specific profiles, according to age groups.
- These new data shed light on the long-term psychosocial correlates of childhood adversities (lower socioeconomic status, perceived social support, and community involvement in adulthood) in the Inuit population of Nunavik.
- The participatory nature of the study based on a two-eyed seeing approach adds to the methodological knowledge in research with Indigenous populations.

What are the key implications for public health interventions, practice, or policy?

- The results inform stakeholders and public health providers about Nunavik Inuit childhood experiences, one of known early determinants of mental and physical health.
- The results highlight the importance of studying and implementing effective culturally informed and safe care and interventions for people in need.
- The results support the relevance of developing primary, secondary, and tertiary prevention interventions, services, and policies aiming to reduce the prevalence and intergenerational transmission of trauma experienced by Inuit populations in Quebec and in the rest of Canada.

### Supplementary information

Below is the link to the electronic supplementary material.

Supplementary file1 (DOCX 23 KB)

## Data Availability

The data presented in this article come from Indigenous populations and thus belong to the communities of Nunavik. Authors cannot share the original data, and requests for access to the data must go through the Data Management Committee of *Qanuilirpitaa*? 2017. However, please note that the authors declare that all the data supporting the findings of this study are available within the article.

## Code availability

The codes and syntax used to perform the analyses presented in the manuscript were written using SPSS software, and were created by the authors, following standard functions and operations. The authors can share the functions and operations that have been used.
