# Supplementary material for: Profiles of childhood adversities in Inuit from Nunavik: description and associations with indicators of socioeconomic characteristics, support, and community involvement
Source: Can J Public Health. 2023 Apr 20;115(Suppl 1):97–113. doi: 10.17269/s41997-023-00750-z (PMC10830971; doi:10.17269/s41997-023-00750-z)
Supplement: Supplementary file 1 — Supplementary file1 (DOCX 23 KB) [file 41997_2023_750_MOESM1_ESM.docx]

Table S1

*Indicators of adverse childhood experiences, residential school attendance, socioeconomic status, support and community involvement*

| Adverse childhood experiences (ACE-Q; Felitti et al., 1998/2019) | |
| --- | --- |
| Sexual violence | “Did an adult or person at least 5 years older than you ever…Touch or fondle you or have you touched their body in a sexual way, or attempt or actually have oral, anal or vaginal intercourse with you?  Dichotomic answer: No (0), Yes (1) |
| Abuse |  |
| Psychological abuse | “Did a parent or other adult in the household often or very often… Swear at you, insult you, put you down, or humiliate you?”  Dichotomic answer: No (0), Yes (1) |
| Physical abuse | “Did a parent or other adult in the household often or very often… Push, grab, slap, or throw something at you?”  Dichotomic answer: No (0), Yes (1) |
| Neglect |  |
| Psychological neglect | “Did you often or very often feel that… No one in your family loved you or thought you were important or special? You didn’t have enough to eat or had to wear dirty clothes?”  Dichotomic answer: No (0), Yes (1) |
| Physical neglect | “Did you often or very often feel that… You didn’t have enough to eat or had to wear dirty clothes?”  Dichotomic answer: No (0), Yes (1) |
| Household stressors |  |
| Witnessing parents separation/divorce | “Were your parents ever separated or divorced?”  Dichotomic answer: No (0), Yes (1) |
| Witnessing domestic violence against mother/stepmother | “Was your mother or stepmother often or very often pushed, grabbed, slapped, or had something thrown at her?”  Dichotomic answer: No (0), Yes (1) |
| Living with someone presenting substance use | “Did you live with anyone who was a problem drinker or alcoholic or who used street drugs?” Dichotomic answer: No (0), Yes (1) |
| Living with someone presenting mental illness or suicidal behaviors | “Was a household member depressed or mentally ill or did a household member attempt suicide?”  Dichotomic answer: No (0), Yes (1) |
| Living with someone who went to prison | “Did a household member go to prison?”  Dichotomic answer: No (0), Yes (1) |
| Residential schools | |
| “Did you ever attend a Residential school?” | Dichotomic answer: No (0), Yes (1) |
| Socioeconomic indicators | |
| Relationship status | “What is your marital status?”:   - In a committed relationship (married or in common law relationship) - Not in a committed relationship (single, separated, divorced, or widowed) |
| Education level | “What is the highest grade you have completed?”   - Secondary school not completed (secondary 4/grade 10 completed or less) - Secondary school completed at least (secondary 5/grade 11 completed or more) |
| Employment | “Which of the following best describes your current status?”   - Not working (support program, housework, retired or pension, unemployment, insurance, parental leave, student) - Working (full time, part time, occasionally, self employed) |
| Income | “What is your best estimate of your total personal income, before taxes and other deductions, from all sources in the past 12 months?”   - Under $20,000 - $20,000 or more |
| Support and community involvement indicators | |
| Perception of support |  |
| Social support | Five statements measuring the perception of four types of social support:   - Positive interactions - Emotional support - Love and affection   Likert scale ranging from 0 (Never), 1 (Rarely), 2 (Sometimes), 3 (Most of the time), 4 (All the time). Total score ranging from 0 to 20, a higher score representing more support. |
| Family cohesion | Questions adapted from the Brief Family Relationship Scale questionnaire:   - In my close family… “there is a feeling of togetherness”, “we really help and support each other”, “we really get along well with each other”, “we spend a lot of time doing things together at home”, “we spend a lot of time doing things together on the land”, “I am proud to be a part of my family”   Likert scale: 0 (Not true), 1 (Somewhat true), 2 (Very true). Total score ranging from 0 to 12, a higher score representing greater family cohesion. |
| Community cohesion | Perception of social cohesion in the community:   - “There is a feeling of togetherness or closeness”, “People help others”, “People can be trusted”, “I feel like I belong”   Likert scale: 0 (Strongly disagree), 1 (Disagree), 2 (Neutral), 3 (Agree), 4 (Strongly agree). Total score ranging from 0 to 24, a higher score representing greater community cohesion. |
| Participation in social activities |  |
| Participation in religious activities | “During the past 12 months, not counting events such as weddings or funerals, how often did you participate in religious activities or attend religious services or meetings?”  Frequency scale: 0 (Never), 1 (Once or a few time/year), 2 (At least once a month).  Dichotomized: 0=Never or Once or a few time/year versus 1=At least once a month. |
| Participation in volunteering and community activities | Frequency of involvement in two types of community activities:   - “Participation in cultural, community or sports events such as festivals, dances, feasts or Inuit games” - “Volunteered for a group, an organization or community event such as a rescue team, church group, feasts, spring clean-up”   Frequency scale: 0 (Never), 1 (Rarely), 2 (Sometimes), 3 (Often), 4 (Always). Total score ranging from 0 to 8, a higher score representing greater involvement. |
| Participation in healing and wellness activities | “In the past 12 months, have you taken part in any activities to promote your own healing or wellness?”  Dichotomic answer: No (0), Yes (1) |
| Practice of traditional activities |  |
| Frequency of going on the land | “From the Spring until now, how often did you go on the land?”  Likert scale: 0 (Never), 1 (Occasionally), 2 (Often).  Dichotomized: 0=Never or Occasionally versus 1=Often. |
| Satisfaction with ability to practice traditional activities | How satisfied are you with your:   - “ability to go out on the land hunting, fishing and berry picking”? - “ability to satisfy country food cravings”? - “ability to communicate with others in Inuktitut”? - “knowledge and skills of cultural and traditional activities, games, and arts”?   Likert scale: 0 (Very dissatisfied), 1 (Dissatisfied), 2 (Neutral), 3 (Satisfied) 4 (Very satisfied). Total score ranging from 0 to 16, a higher score representing higher satisfaction with ability to practice traditions. |

Table S2

*Goodness-of-fit statistics and classification coefficients*

| Number of clusters | AIC | ∆AIC | BIC | ∆BIC | Entropy | Smallest unweighted cluster *n* (%) |
| --- | --- | --- | --- | --- | --- | --- |
| 18 to 49-year-olds | | | | |  |  |
| 1 | 9083.85 | - | 9130.30 | - | - | 781 (100.0) |
| 2 | 8243.71 | -840.14 | 8341.58 | -788.72 | 0.775 | 281 (35.9) |
| **3** | **8139.55** | **-104.16** | **8288.68** | **-52.9** | 0.687 | 206 (26.4) |
| 4 | 8069.64 | -69.91 | 8270.05 | -18.63 | 0.735 | 62 (7.9) |
| 5 | 8043.43 | -26.21 | 8295.15 | 25.1 | 0.766 | 34 (4.4) |
| 50-year-olds and over who experienced residential schooling | | | | |  |  |
| 1 | 926.49 | - | 950.95 | - | - | 112 (100.0) |
| **2** | **834.70** | **-91.79** | **886.36** | **-64.59** | 0.878 | 22 (19.9) |
| 3 | 835.13 | 0.43 | 913.94 | 27.58 | 0.927 | 3 (2.2) |
| 4 | 837.85 | 2.72 | 943.87 | 29.93 | 0.970 | 11 (9.8) |
| 5 | 843.47 | 5.62 | 976.68 | 32.81 | 0.967 | 2 (1.8) |
| 50-year-olds and over who did not experience residential schooling | | | | |  |  |
| 1 | 1821.17 | - | 1851.55 | - | - | 216 (100.0) |
| **2** | **1583.13** | **-238.04** | **1647.26** | **-204.29** | 0.886 | 49 (22.8) |
| 3 | 1577.55 | -5.58 | 1675.43 | 28.17 | 0.913 | 24 (11.1) |
| 4 | 1576.56 | -0.99 | 1708.19 | 32.76 | 0.937 | 7 (3.2) |
| 5 | 1579.94 | 3.38 | 1745.33 | 37.14 | 0.943 | 7 (3.2) |

*Note.:* AIC, Akaike's Information Criterion; BIC, Bayesian Information Criterion.

Bolded values indicate best fit for each group.
